# Systematic review of prophylactic antibacterial agents for radiation-induced oral mucositis in head and neck cancer

**DOI:** 10.1007/s00520-026-10439-x

**Published:** 2026-02-23

**Authors:** Daniel S. Alicea, Mark Hans, Zahidul Islam, Rachel Schwartz, Thomas J. Ow, Vikas Mehta, Madhur Garg, Beth N. McLellan, Rafi Kabarriti

**Affiliations:** 1 Division of Dermatology, Department of Medicine, Albert Einstein College of Medicine, Bronx, NY USA; 2 Department of Radiation Oncology, Montefiore Einstein Comprehensive Cancer Center, Bronx, NY USA; 3 D. Samuel Gottesman Library, Albert Einstein College of Medicine, Bronx, NY USA; 4 Department of Otorhinolaryngology-Head and Neck Surgery, Montefiore Medical Center/Albert Einstein College of Medicine, Bronx, NY USA; 5 Albert Einstein College of Medicine, Bronx, NY USA

**Keywords:** Systematic review, Radiation induced oral mucositis, Radiotherapy, Head and neck cancer, Chemoradiotherapy

## Abstract

**Objective:**

To examine the use of treatments with antibacterial properties as prophylaxis prior to radiotherapy (RT), either alone or in combination with chemotherapy (CT), to prevent and reduce radiation-induced oral mucositis (RIOM) in patients with head and neck cancer (HNC).

**Data sources:**

A systematic search following PRISMA guidelines was conducted across PubMed, Embase, Web of Science, and the Cochrane Library to identify relevant studies published in English through March 2025.

**Review methods:**

Eligible studies assessed prophylactic antibacterial interventions aimed at preventing RIOM.

**Results:**

From 86 retrieved citations, 9 articles met inclusion criteria. Antibacterial agents assessed included polymyxin, tobramycin, amphotericin (PTA), povidone iodine, SAMITAL, and Nigella sativa (NS). Evidence supporting povidone iodine, PTA, and SAMITAL was inconclusive or failed to demonstrate statistically significant reductions in RIOM severity. Several studies reported discordant findings, with statistically significant improvements in patient-reported symptoms or quality-of-life measures despite nonsignificant clinician-assessed scores. NS demonstrated potential benefits in reducing RIOM incidence and severity compared with standard of care and other antibacterial agents.

**Conclusion:**

The systematic review highlights limited and inconsistent evidence supporting antibacterial prophylaxis for preventing and reducing RIOM severity in patients with HNC undergoing RT. Discrepancies between patient-reported outcomes and clinical-assessed grading suggest some treatments may provide symptomatic benefit not captured by traditional scoring systems. NS mouthwashes showed preliminary promise; however, evidence remains insufficient to establish superiority, and safety and regulatory concerns are persistent, particularly in immunosuppressed patients. Given the role of bacterial colonization and microbial dysbiosis in RIOM pathogenesis, larger, well-designed clinical trials with rigorous safety evaluations are warranted to investigate bacterial-directed preventive therapies.

## Introduction

Radiotherapy (RT), alone or combined with chemotherapy (CT) remains a main treatment modality for head and neck cancer (HNC) [1]. Radiation-induced oral mucositis (RIOM) is one of the most common acute toxicities associated with RT [2]. RIOM refers to inflammation of the mucosal lining epithelium caused by cytotoxic treatments, which occurs in the majority of patients receiving RT, and almost universally in patients receiving chemoradiotherapy (CRT) [3], with estimates of 66–85% of patients suffering from Common Terminology Criteria for Adverse Events (CTCAE) grade 3–4 RIOM [4]. RIOM has various presentations often manifesting with white and/or yellow patches, ulceration, mucosal atrophy, erythema, edema and bleeding [5]. RIOM can be divided into five stages: initiation, response to primary damage, signal amplification, ulceration and healing. The pathogenesis starts with exposure to reactive oxygen species leading to cell damage, activation of NF-kB, expression of IL-1, IL-6 and TNF-alpha, and production of metalloproteinases leading to cell damage and death [6]. Oral mucosa destruction, particularly in the ulceration stage, fosters microorganism colonization with viruses, bacteria or fungi, enhancing the already present inflammation and increasing lesion severity [7]. Although historically our understanding of the pathogenesis of radiation-induced toxicities has focused on cellular damage and resulting inflammation, newer evidence suggests an important role of the local microbiome both at baseline and as it changes during treatment [8]. In RIOM, it has been proposed that baseline microbial dysbiosis with less abundant oral bacteria increases inflammation and development of RIOM [9]. There is a crucial need to better understand and manage RIOM and thus prevent resulting dysphagia, weight loss, malnutrition, dysarthria and superinfections [10].

These acute toxicities can be detrimental to quality of life. In addition, they may adversely impact the oncologic treatment plan. There are many treatments to choose from for the prevention and management of RIOM, yet there is still no gold-standard protocol [11]. The most effective strategies to reduce RIOM remain unclear. Different techniques have been utilized including, but not limited to, intensive oral care, anti-microbial, anti-inflammatory, and cytoprotective agents, nutritional supplements, biostimulants, and/or natural and homeopathic agents [12]. Despite their clinical use, specific agents such as chlorhexidine and compounded “magic mouthwash” formulations have not been supported by published evidence for the prevention of RIOM given the insufficient and inconsistent data in significantly reducing the incidence or severity of RIOM [13].

Limited studies exist that discuss the role of antimicrobial prophylaxis in preventing inflammatory pathologies such as RIOM. A review of the literature showed that the most up-to-date meta-analyses of preventive intervention possibilities in RIOM were published in 2006 [13]. Over the past twenty years, the study of newer antimicrobial agents targeting RIOM has progressed, and improved documentation and scoring of these toxicities have enabled physicians to more accurately record RIOM grading. Some of these newer agents, particularly natural compounds, have multi-modal antimicrobial effects with antibacterial, antifungal, and antiviral properties. Given the particularly prominent role of bacterial colonization and dysbiosis in the development and severity of RIOM, bacterial decolonization (BD)—the targeted elimination or reduction of potentially pathogenic bacteria from specific body sites—has been proposed as a strategy to prevent or mitigate these toxicities [26]. Thus, this paper provides an updated literature review on the use of antibacterial treatments to prevent or mitigate RIOM. Findings from this review may support the need for future studies and clinical trials to investigate the role of BD prior to RT in reducing RIOM incidence and severity in patients with HNC.

## Methods

This systematic review searched for prophylactic antibacterial treatments for the prevention of RIOM in patients with head and neck cancer using the Preferred Reporting Items for Systematic Reviews and Meta-analyses (PRISMA) guidelines. Searches were conducted on March 5th, 2025, across PubMed, Embase, Web of Science, and the Cochrane Library. Search results were limited to adult (ages ≥ 18 years) humans and English-language studies. The protocol was registered in the International Prospective Register of Systematic Reviews database (PROSPERO registration number: CRD42023462194).

A search for all peer-reviewed articles was performed with three collections of keywords that were constructed, consisting of: “head and neck neoplasms” and numerous substitute phrases that refer to the same set of conditions (component A); “Anti-Bacterial Agents” and numerous substitute phrases that refer to such agents (component B); and “radiation induced mucositis” and numerous substitute phrases that refer to these conditions (component C). The keywords were then entered into our search of article titles and abstracts, and the results were organized by the component, or combination of components, that were matched. The search strategy included only terms relating to the study.

The criteria for determining study eligibility were defined with regard to participants, interventions, comparator, outcomes, and study design (Table 1). Studies must have utilized a clinical grading system to evaluate the grade/severity of RIOM. Studies that did not have these inclusion criteria were excluded.

**Table 1:** Overview of the PICOS eligibility criteria

Populations	Inclusion: Adults with head and neck cancer receiving RT ± CT Exclusion: Adolescents (under 18 years of age), adults diagnosed with any other cancer and adults diagnosed with head and neck cancer not receiving RT ± CT
Intervention	Patients with head and neck cancer who received bacterial decolonization prior to and during RT ± CT
Comparator	Patients with head and neck cancer who received placebo “bacterial decolonization” or standard of care prior to and during RT ± CT
Outcomes	Severity of RIOM
Study design	Inclusion: Randomized and non-randomized controlled trials, clinical trials, cohort studies Exclusion: Books and documents, conference abstracts, non-English studies

RT-Radiotherapy; CT-Chemotherapy; RIOM-Radiation Induced Oral Mucositis

Two authors (DSA and MH) independently reviewed all articles, selected studies, and extracted data. A descriptive synthesis approach was conducted to extract, combine, and evaluate the data of the individual studies. A meta-analysis was not performed due to the significant clinical and methodological heterogeneity between studies.

## Results

**Fig. 1:**
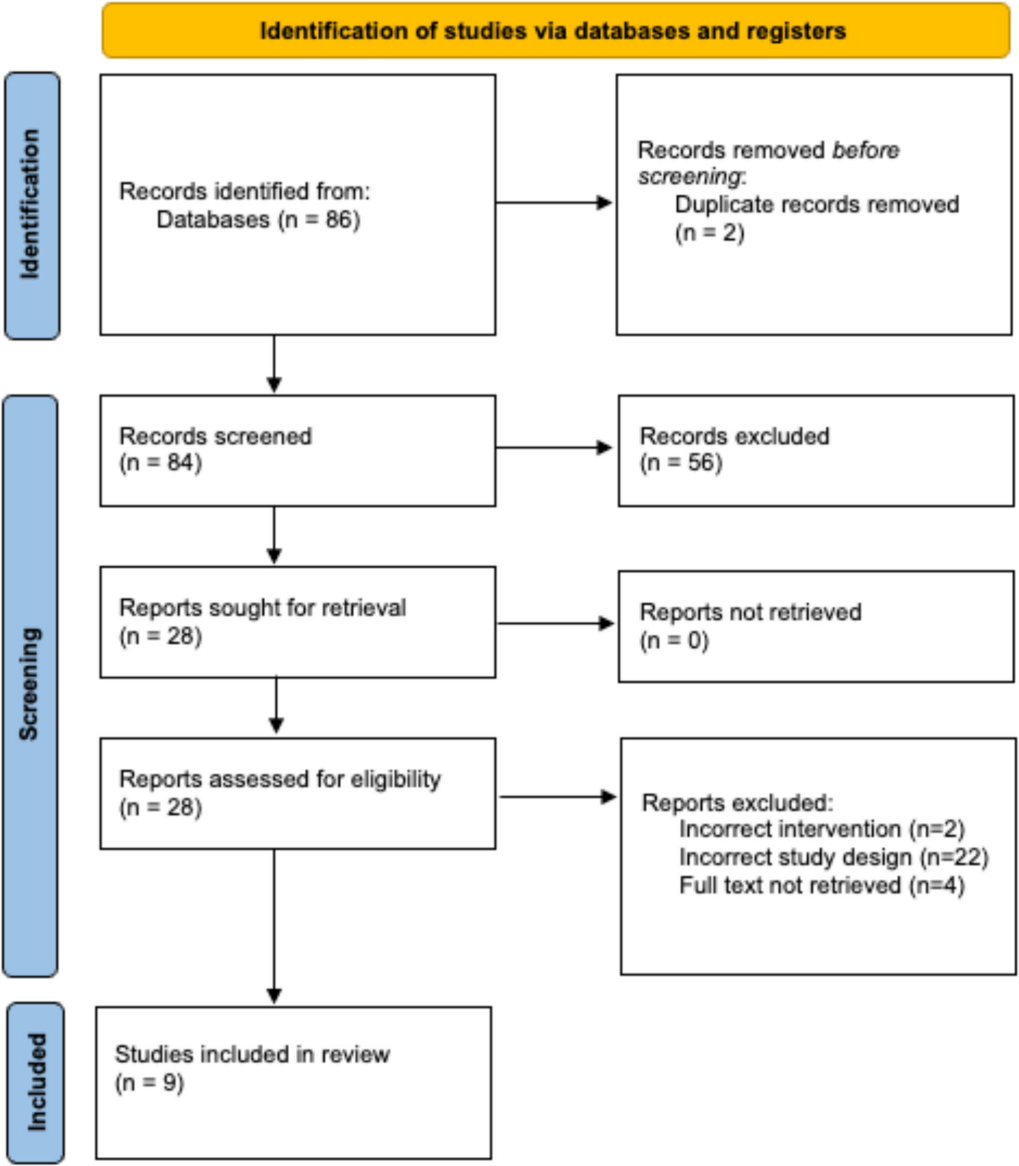
PRISMA flow diagram illustrating the process of study selection

Figure 1 shows the PRISMA flow diagram for the selection of studies [14]. In total, 86 citations were retrieved from the initial search of PubMed, Embase, Web of Science, and the Cochrane Library databases. After removing 2 duplicates, 84 articles were screened based on titles and abstracts, of which 28 articles met criteria for full-text review. Of these, 8 studies were excluded: 2 had an incorrect intervention, 22 had an incorrect study design, and 4 full texts could not be retrieved. No additional studies were identified after screening the reference lists of relevant articles. The remaining 9 studies and their characteristics are listed in Table 2.

**Table 2:** Study characteristics

Author	Type of study	Criteria for assessment	Follow-up duration	Total population; Head and Neck Tumor type	Radiotherapy dosage	Intervention and formulation	Outcome
Ameen et al. [22]	Prospective open-label RCT	RTOG	6–7 weeks	40; 19 larynx, 4 nasal cavity, 4 oral cavity, 9 pharynx	Dose delivered in Gy ranged from 60–70 Gy	20 patients received NS oil mouthwash while 20 patients received control Magic mouthwash	NS significantly reduced RTOG grade and RIOM severity after 2–4 weeks of RT compared with no treatment
Fasanaro et al. [25]	Phase 2, double-blinded RCT	WHO, OAG, OMAS	19 weeks	116; 25 oral cavity, 56 oropharynx, 16 hypopharynx, 19 larynx	Median dose delivered in Gy is 66	59 patients received SAMITAL granules while 57 patients received placebo	SAMITAL did not significantly reduced the incidence of severe RIOM despite a lower rate of RIOM and significantly better quality of life existing in the SAMITAL group compared to placebo
Namuangchan et al. [20]	Double-blinded RCT	OMAS	12 weeks	20; 4 oral cavity, 6 oropharynx, 6 nasopharynx, 2 nasal cavity	Median radiation dose in Gy is 70	10 patients received IS in the experimental group while 10 patients received NSS in the control group	OMAS was not statistically significant in the experimental group compared to the control group
Okuno et al. [15]	Prospective, double-blinded RCT	WHO	9 weeks	112 HNSCC	30–44.99 Gy – 1 patient; 45.00–50.00–8 patients; 50.01–60.00–35 patients; > 60.00–68 patients	54 patients received PTA lozenge/tablet while 58 patients received a placebo lozenge/tablet	Nonabsorbable antibiotic lozenge decreased patient-reported RIOM to a modest degree, but not compelling enough to recommend treatment as part of standard practice
Pawar et al. [24]	Randomized, placebo-controlled, single-blind phase II study	WHO	7 weeks	30; 9 tongue, 1 palate, 2 mandible, 2 alveolus, 1 epiglottis, 6 cheek, 1 larynx, 1 tonsil, 1 vocal cord, 3 unspecified	Total dose of 60 Gy	20 patients received SAMITAL in the experimental group and 10 patients received placebo	SAMITAL significantly decreased the severity of RIOM compared to placebo
Rahn et al. [19]	Prospective, open-label RCT	WHO	14 weeks	40; 17 oral mucosa, 4 maxillary sinus, 7 oropharynx, 7 hypopharynx, 5 other	Total dose of 71.3 Gy	20 patients received IS in the experimental group while 20 patients received sterile water rinse in the control group	RIOM incidence, severity and duration was significantly reduced in the IS group compared to the control group
Stokman et al. [17]	Double-blinded RCT	WHO	5 weeks	65; 40 oral cavity, 18 oropharynx, 2 hypopharynx, 5 unknown primary	Dose of at least 50 Gy	33 patients received PTA lozenges and 32 patients received the placebo lozenges	RIOM score did not differ between the two groups
Kannarunimit et al. [18]	Prospective, single-blinded RCT	OMAS, NCI-CTCAE v5.0	12 weeks	71; Primary disease site was the oral cavity and oropharynx (66.3%) followed by the nasopharynx, hypopharynx, and supraglottic area (33.7%)	Mean dose was 48.9 Gy	37 patients received benzydamine hydrochloride as standard of care in the control group and 34 patients received povidone iodine in the experimental group	Povidone-iodine correlated with less RIOM and a significant lower incidence of grade III-IV CTCAE RIOM compared to benzydamine hydrochloride
Wijers et al. [16]	Placebo-controlled double-blinded RCT	WHO	8 weeks	77; 23 oral cavity, 26 oropharynx, 3 nasopharynx, 4 hypopharynx, 4 larynx, 2 maxillary sinus, 11 salivary glands, 4 miscellaneous	14 patients received 46–50 Gy, 22 patients received 60 Gy, 41 patients received 70 Gy	39 patients received PTA lozenges and 38 patients received the placebo lozenges	No significant difference for the objective and subjective RIOM was observed between the two study arms

RCT-randomized controlled trial; RTOG-Radiation Therapy Oncology Group; NS-Nigella sativa; WHO-World Health Organization; OAG-Oral Assessment Guide; OMAS-Oral Mucositis Assessment Scale; RIOM-Radiation Induced Oral Mucositis; PTA-Polymyxin, Tobramycin, and Amphotericin; NCI-CTCAE-National Cancer Institute-Common Terminology Criteria for Adverse Events; IS-Iodine Solution; NSS-Normal Saline Solution; HNSCC – Head and Neck Squamous Cell Carcinoma

### Polymyxin, tobramycin, amphotericin

In response to the absence of an established prophylactic method to decrease RIOM, three prospective studies [15–17] examined the use of PTA (Polymyxin and Tobramycin—nonabsorbable antibiotics; Amphotericin – polyene antifungal) in patients undergoing RT to the HNC region. It has been suggested that the selective elimination of the aerobic Gram-negative bacteria (AGNB) and yeast species using antibacterial lozenges may lead to a reduction of RIOM severity. Okuno et at. [15] did not observe any significant trends in RIOM scores measured by health care providers. However, patient-reported RIOM scores and duration of severe grade (grade 3–4) RIOM were statistically lower in the experimental group. With limited studies examining the prospective analysis of changes in the oral microbiome, Stokman and Wijers et at. [16, 17] noted microbiologic counts/changes of the oral flora before and after RIOM with PTA prophylaxis. However, both trials showed that PTA prophylaxis had a minimal effect on the RIOM grade despite selective elimination of AGNB and Candida species, thus suggesting against its use in preventing RIOM.

### Povidone iodine

A recent 2023 RCT study [18] proposed the use of povidone-iodine mouth wash to prevent RIOM, based on its broad-spectrum antiseptic and anti-inflammatory properties, leading to a decrease in bacterial infection, reduction of proinflammatory cytokines, and promotion of healing signals. Results showed superiority of 0.1% povidone-iodine in both Oral Mucositis Assessment Score (OMAS) and grade 3/4 NCI-CTCAE toxicity assessments compared to standard of care benzydamine hydrochloride (a nonsteroidal anti-inflammatory medication) with the incidence of grade III-IV CTCAE in 26.5% of patients in the povidone-iodine cohort compared to 51.4% of patients in the benzydamine hydrochloride cohort (*p*-value = 0.032). A similar 1997 RCT also reported a significant reduction of RIOM incidence in the treatment arm compared to the control arm [19]. On the other hand, a 2023 prospective, double-blind, RCT was conducted on 20 HNC patients with results showing no statistically significant difference in the prevention of RIOM between the two groups and encouraged a larger number of participants for future studies [20]. This was consistent with the results of a larger multicenter RCT studying 132 patients which found no difference between mouth rinsing with povidone-iodine and standard of care.

### SAMITAL

A phase 2 RCT clinical trial investigated the use of SAMITAL in reducing the incidence of severe RIOM. SAMITAL is a botanical drug composed of *V. myrtillus*, *M. cordata* dried fruits, and *E.* *angustifolia* dried roots [21]. These standardized botanical extracts are dispersed in water in a gel-like suspension with antibacterial, antifungal, antiviral, and anti-inflammatory properties [21]. The study results did not show a significant superiority of SAMITAL compared with the placebo in reducing the incidence of RIOM.

### Nigella Sativa (NS)

A prospective open-label clinical study [22] aimed to evaluate the anti-inflammatory effect of NS oil as a mouthwash in reducing RIOM in patients with HNC. NS, or black cumin, is a natural compound composed of *Nigella sativa* *Ranunculaceaeis*. The active ingredient, thymoquinone, has antimicrobial, anti-inflammatory, and antioxidant properties. Patients using the NS oil had a significantly lower RTOG grade than those in the control group at week 4 until the end of treatment (*p*-value < 0.05), thus posing it as a potential prophylactic treatment for the prevention of RIOM.

## Discussion

RT ± CT leads to RIOM in almost every patient with HNC [23]. RIOM can significantly impact a patient’s prognosis and overall quality of life. When severe, RIOM can lead to treatment interruption, dose reduction, or suspension of cancer-directed treatments, ultimately impacting oncologic outcomes. Because bacterial colonization is an independent risk factor for the development of RIOM [9], BD may assist with preventing RIOM development or progression. Although numerous agents have been studied, the overall impact on RIOM has been mixed, and an ideal strategy has not materialized.

Povidone-iodine, a broad-spectrum antiseptic agent, was among the strategies identified in this review. Given the insufficient and conflicting evidence, no clinical guidelines have been established for povidone-iodine prophylaxis. Other studies listed in the review investigated the effects of a combination antimicrobial treatment, PTA, in a lozenge for selective elimination of aerobic Gram-negative bacteria and yeast. Based on our analysis, several randomized placebo-controlled trials had results that universally did not recommend PTA as a preventive measure for RIOM. This was consistent with a 2006 meta-analysis that included five studies of PTA prevention of RIOM in HNC patients; outcomes showed no significant effect on the prevention of RIOM [13]. Thus, the discussed body of evidence continues to support against the use of these combined antimicrobial treatment for OM prevention.

Natural products have been utilized in experimental and clinical settings to treat RIOM, which have, in turn, prompted investigation of these products as prophylactic agents for RIOM. These agents, particularly SAMITAL and NS, possess multiple antimicrobial properties, including antifungal and antiviral activity. While these compounds are not strictly antibacterial, prior evidence has explored their importance as possible antibacterial targets in reducing and treating RIOM. Future studies may further clarify the relative contributions of antibacterial versus other antimicrobial effects in reducing and treating RIOM.

SAMITAL, a relatively new botanical drug extract utilized within the last decade, has been designed with the aim of treating RIOM. Previous studies reported relevant reductions in RIOM grade with decreased pain and improvement in quality of life in patients treated with SAMITAL compared to the placebo group [24]. However, a 2022 phase 2 clinical trial showed that SAMITAL did not significantly reduce the incidence of severe RIOM, but the overall lower rate of RIOM led to a significantly better quality of life and thus posed a clinical benefit [25]. Considering these clinical trials had similar study designs, SAMITAL's effects should be further investigated on a larger sample size.

Given its use in reducing RTOG grade and RIOM severity of HNC patients, NS oil mouthwash presents as a prophylactic and treatment regimen that could be utilized in the clinical setting. However, this is limited to a single small-scale study [22] with the need for further studies with larger sample sizes to enable and draw more meaningful conclusions. Furthermore, while NS oil mouthwash may offer a novel approach, its application raises significant safety concerns. As a natural product derived from Nigella sativa Ranunculaceae, it lacks regulatory approval for cancer-related indications and is often used off-label or in unregulated formulations. This is particularly concerning for immunosuppressed patients with ulcerated mucosa, who may be at increased risk for bacteremia or adverse systemic effects. These risks highlight the critical importance of pharmacovigilance and the need for well-designed, large-scale clinical trials to thoroughly evaluate its safety and efficacy.

A 2023 phase 2/3 RCT determined the efficacy of BD with intranasal mupirocin ointment and chlorhexidine gluconate cleanser in reducing the severity of acute radiation dermatitis (ARD) in patients with breast and HNC receiving RT [26]. Their prior study demonstrated that SA had proinflammatory and pathogenic properties in skin diseases, with SA colonization serving as an independent risk factor for the development of severe grade II or higher ARD. The present study demonstrated that the mean (SD) ARD grade was significantly lower in the experimental cohort compared to patients in the standard of care cohort (*p*-value = 0.02), with 0% of patients in the experimental group demonstrating grade 2 or higher ARD compared to 23.7% of patients in the control group. Given the similarities in pathogenesis and toxicities of ARD and RIOM, BD with this antibacterial regimen should continue to be explored as a potential therapeutic prophylactic agent in preventing and reducing RIOM.

A 2025 single-center, open-label, phase 3 RCT evaluated the efficacy of BD with intranasal mupirocin ointment in alleviating severe (grade ≥ 3) RIOM in patients with nasopharyngeal cancer receiving CRT [27]. Because the oral and nasal cavities share bacterial species such as *Staphylococcus aureus*, bacterial overgrowth during CRT serves as a potential risk factor for RIOM. Mupirocin nasal ointment could decolonize these bacteria, thereby lowering the risk of severe RIOM. The present study demonstrated that patients in the BD group had a 52% relative reduction in the risk of developing severe RIOM compared with those receiving standard of care (RR, 0.48; 95% CI, 0.31–0.74; *p*-value = < 0.001). The results indicate that intranasal mupirocin ointment serves as a possible cost-effective BD agent in reducing RIOM. Further multicenter studies are needed to support these findings and contribute to the microbial management of radiation-related complications in this patient population.

While antibacterial/antimicrobial prophylaxis was the focus of this systematic review, there are other modalities that are beyond the scope of this article but are imperative and have been studied as potential prevention strategies for RIOM including: probiotics, anti-inflammatory drug subgroups, low intensity laser therapy and cryotherapy. Future directions focusing on evidence-based clinical practice guidelines for RIOM with study groups such as the Mucositis Study Group of the Multinational Association of Supportive Care in Cancer/International Society of Oral Oncology (MASCC/ISOO) are warranted to explore various treatments and ultimately provide updated and generalized clinical recommendations.

## Limitations

Our systematic review has some limitations. Three of the desired full texts could not be retrieved as one was in a non-English language journal despite our initial exclusion criteria of non-English studies, and 2 additional articles were published as abstracts with no full text option available. Although the determination of which articles to include in the review was subject to bias, re-review of these articles helped mitigate this bias.

## Conclusion

This systematic review demonstrates that a wide variety of agents have been evaluated for the prevention of RIOM. To date, it can be concluded that, for some agents, the evidence was insufficient to support some antibacterial interventions as prophylaxis for preventing RIOM. The oral microbiome plays a role in RIOM, with antibiotic prophylactic strategies such as Povidone-iodine, PTA and SAMITAL yielding mixed results, while other antibiotic prophylactic strategies, such as NS, demonstrate preliminary promise, though lack regulatory approval and raise significant safety concerns, particularly for immunocompromised patients. Importantly, however, some studies reported discordant findings, wherein improvements in patient-reported symptoms or quality-of-life measures were observed despite the absence of significant changes in healthcare provider-reported outcomes. Our review identifies key gaps in our current knowledge regarding strategies to address RIOM. Further studies are required to develop an optimal strategy with a focus on evaluating novel therapies with comprehensive safety monitoring to address the inconsistencies in the already existing treatment modalities for the prevention of RIOM.

## Data Availability

No datasets were generated or analyzed during the current study.

## References

[1] Chow LQM (2020) Head and neck cancer. N Engl J Med 382(1):60–72. 10.1056/NEJMra1715715 31893516 10.1056/NEJMra1715715

[2] Elad S, Cheng KKF, Lalla RV et al (2020) MASCC/ISOO clinical practice guidelines for the management of mucositis secondary to cancer therapy. Cancer 126(19):4423–4431. 10.1002/cncr.33100 32786044 10.1002/cncr.33100 PMC7540329

[3] Elting LS, Cooksley CD, Chambers MS, Garden AS (2007) Risk, outcomes, and costs of radiation-induced oral mucositis among patients with head-and-neck malignancies. Int J Radiat Oncol Biol Phys 68(4):1110–1120. 10.1016/j.ijrobp.2007.01.053 17398022 10.1016/j.ijrobp.2007.01.053

[4] Sanguineti G, Gunn GB, Parker BC, Endres EJ, Zeng J, Fiorino C (2011) Weekly dose-volume parameters of mucosa and constrictor muscles predict the use of percutaneous endoscopic gastrostomy during exclusive intensity-modulated radiotherapy for oropharyngeal cancer. Int J Radiat Oncol Biol Phys 79(1):52–59. 10.1016/j.ijrobp.2009.10.057 20418027 10.1016/j.ijrobp.2009.10.057

[5] Lalla RV, Bowen J, Barasch A et al (2014) MASCC/ISOO clinical practice guidelines for the management of mucositis secondary to cancer therapy. Cancer 120(10):1453–1461. 10.1002/cncr.28592 24615748 10.1002/cncr.28592 PMC4164022

[6] Pulito C, Cristaudo A, Porta C et al (2020) Oral mucositis: the hidden side of cancer therapy. J Exp Clin Cancer Res 39(1):210. 10.1186/s13046-020-01715-7 33028357 10.1186/s13046-020-01715-7 PMC7542970

[7] Lalla RV, Sonis ST, Peterson DE (2008) Management of oral mucositis in patients who have cancer. Dent Clin North Am 52(1):61–77. 10.1016/j.cden.2007.10.002 18154865 10.1016/j.cden.2007.10.002 PMC2266835

[8] Kost Y, Rzepecki AK, Deutsch A et al (2023) Association of *Staphylococcus aureus* colonization with severity of acute radiation dermatitis in patients with breast or head and neck cancer. JAMA Oncol 9(7):962–965. 10.1001/jamaoncol.2023.0454 37140927 10.1001/jamaoncol.2023.0454 PMC10160990

[9] Hajishengallis G, Darveau RP, Curtis MA (2012) The keystone-pathogen hypothesis. Nat Rev Microbiol 10(10):717–725. 10.1038/nrmicro2873 22941505 10.1038/nrmicro2873 PMC3498498

[10] Sanguineti G, Rao N, Gunn B, Ricchetti F, Fiorino C (2013) Predictors of PEG dependence after IMRT±chemotherapy for oropharyngeal cancer. Radiother Oncol 107(3):300–304. 10.1016/j.radonc.2013.05.021 23773408 10.1016/j.radonc.2013.05.021

[11] Diaz-Sanchez RM, Pachón-Ibáñez J, Marín-Conde F, Rodríguez-Caballero Á, Gutierrez-Perez JL, Torres-Lagares D (2015) Double-blind, randomized pilot study of bioadhesive chlorhexidine gel in the prevention and treatment of mucositis induced by chemoradiotherapy of head and neck cancer. Med Oral Patol Oral Cir Bucal 20(3):e378–e385. 10.4317/medoral.20338 25662553 10.4317/medoral.20338 PMC4464927

[12] Gugnacki P, Sierko E (2021) Is there an interplay between oral microbiome, head and neck carcinoma and radiation-induced oral mucositis? Cancers (Basel). 10.3390/cancers13235902 34885015 10.3390/cancers13235902 PMC8656742

[13] Stokman MA, Spijkervet FK, Boezen HM, Schouten JP, Roodenburg JL, de Vries EG (2006) Preventive intervention possibilities in radiotherapy- and chemotherapy-induced oral mucositis: results of meta-analyses. J Dent Res 85(8):690–700. 10.1177/154405910608500802 16861284 10.1177/154405910608500802

[14] Page MJ, McKenzie JE, Bossuyt PM et al (2021) The PRISMA 2020 statement: an updated guideline for reporting systematic reviews. BMJ 372:n71. 10.1136/bmj.n71 33782057 10.1136/bmj.n71 PMC8005924

[15] Okuno SH, Foote RL, Loprinzi CL et al (1997) A randomized trial of a nonabsorbable antibiotic lozenge given to alleviate radiation-induced Mucositis. Cancer 79(11):2193–2199. 10.1002/(sici)1097-0142(19970601)79:11<2193::Aid-cncr18>3.0.Co;2-r 9179067 10.1002/(sici)1097-0142(19970601)79:11<2193::aid-cncr18>3.0.co;2-r

[16] Wijers OB, Levendag PC, Harms ERE et al (2001) Mucositis reduction by selective elimination of oral flora in irradiated cancers of the head and neck: a placebo-controlled double-blind randomized study. Int J Radiat Oncol Biol Phys 50(2):343–352. 10.1016/s0360-3016(01)01444-4 11380220 10.1016/s0360-3016(01)01444-4

[17] Stokman MA, Spijkervet FKL, Burlage FR et al (2003) Oral mucositis and selective elimination of oral flora in head and neck cancer patients receiving radiotherapy: a double-blind randomised clinical trial. Br J Cancer 88(7):1012–1016. 10.1038/sj.bjc.6600824 12671696 10.1038/sj.bjc.6600824 PMC2376383

[18] Kannarunimit D, Chotirut A, Prayongrat A et al (2023) A prospective randomized study comparing the efficacy between povidone-iodine gargling and benzydamine hydrochloride for mucositis prevention in head and neck cancer patients receiving concurrent chemoradiotherapy. Heliyon 9(4):8. 10.1016/j.heliyon.2023.e15437 10.1016/j.heliyon.2023.e15437 PMC10161604 37151677

[19] Rahn R, Adamietz IA, Boettcher HD, Schaefer V, Reimer K, Fleischer W (1997) Povidone-iodine to prevent mucositis in patients during antineoplastic radiochemotherapy. Dermatology 195(Suppl 2):57–61. 10.1159/000246032 9403257 10.1159/000246032

[20] Namuangchan Y, Chailertwanich O, Susinsamphan S et al (2023) Prophylaxis of oral mucositis with iodine solution during concurrent chemoradiation of head and neck cancer: preliminary results of a double-blind, randomized controlled trial. Asian Pac J Cancer Prev 24(7):2445–2454. 10.31557/apjcp.2023.24.7.2445 37505779 10.31557/APJCP.2023.24.7.2445 PMC10676485

[21] Morazzoni P, Petrangolini G, Bombardelli E, Ronchi M, Cabri W, Riva A (2013) SAMITAL®: a new botanical drug for the treatment of mucositis induced by oncological therapies. Future Oncol 9(11):1717–1725. 10.2217/fon.13.165 24156331 10.2217/fon.13.165

[22] Ameen HAM, Mohammed MO, Ahmed KM, Ali RHG, Saeed KA, Hussain SA (2019) Anti-inflammatory effect of *Nigella Sativa* oil on chemoradiation-induced oral mucositis in patients with head and neck cancers. Int J Curr Pharm Res 11(5):58–64. 10.22159/ijcpr.2019v11i5.35704

[23] Mehta V, Sarode GS, Obulareddy VT et al (2023) Clinicopathologic profile, management and outcome of sinonasal ameloblastoma-a systematic review. J Clin Med. 10.3390/jcm12010381 36615180 10.3390/jcm12010381 PMC9821057

[24] Pawar D, Neve RS, Kalgane S et al (2013) SAMITAL® improves chemo/radiotherapy-induced oral mucositis in patients with head and neck cancer: results of a randomized, placebo-controlled, single-blind phase II study. Support Care Cancer 21(3):827–834. 10.1007/s00520-012-1586-5 22945882 10.1007/s00520-012-1586-5

[25] Fasanaro E, Del Bianco P, Groff E et al (2022) Role of SAMITAL in the prevention and treatment of chemo-radiotherapy-induced oral mucositis in head and neck carcinoma: a phase 2, randomized, double-blind, placebo-controlled clinical trial (ROSAM). Cancers. 10.3390/cancers14246192 36551677 10.3390/cancers14246192 PMC9776559

[26] Kost Y, Deutsch A, Mieczkowska K et al (2023) Bacterial decolonization for prevention of radiation dermatitis: a randomized clinical trial. JAMA Oncol 9(7):940–945. 10.1001/jamaoncol.2023.0444 37140904 10.1001/jamaoncol.2023.0444 PMC10160991

[27] Liao Z, Xiong X, Zhao L, Zhang Z, Guan C, Zhang L, Zhong F, Rao J, Wang X, Xiao Y, Gong X, Huang SH, Li J, Lu T (2025) Bacterial decolonization with mupirocin ointment for acute radiation oral mucositis prevention: a phase 3 randomized clinical trial. JAMA Oncol 11(10):1141–1149. 10.1001/jamaoncol.2025.2361 40773220 10.1001/jamaoncol.2025.2361 PMC12332761

