# Correction to: Systematic review of prophylactic antibacterial agents for radiation‑induced oral mucositis in head and neck cancer

**DOI:** 10.1007/s00520-026-10806-8

**Published:** 2026-06-04

**Authors:** Daniel S. Alicea, Mark Hans, Zahidul Islam, Rachel Schwartz, Thomas J. Ow, Vikas Mehta, Madhur Garg, Beth N. McLellan, Rafi Kabarriti

**Affiliations:** 1 Division of Dermatology, Department of Medicine, Albert Einstein College of Medicine, Bronx, NY USA; 2 Department of Radiation Oncology, Montefiore Einstein Comprehensive Cancer Center, Bronx, NY USA; 3 D. Samuel Gottesman Library, Albert Einstein College of Medicine, Bronx, NY USA; 4 Department of Otorhinolaryngology‑Head and Neck Surgery, Montefiore Medical Center/Albert Einstein College of Medicine, Bronx, NY USA; 5 Albert Einstein College of Medicine, Bronx, NY USA

**Correction to: Supportive Care in Cancer (2026) 34:234**

10.1007/s00520-026-10439-x

The second author's correct family name should be "Hans" instead of "Han." The proper name for the second author is Mark Hans.

The publisher regrets this error.

The original article has been corrected.

